# Comparative Clinical Study of the Effect of Nigella Sativa Oil on Soft Tissue Healing and Inflammation Reduction Compared to Eugenol in the Context of Dry Socket

**DOI:** 10.7759/cureus.35375

**Published:** 2023-02-23

**Authors:** Mohamad Alabdullah, Zafin Z Kara Beit, Asaad Shehada

**Affiliations:** 1 Department of Oral and Maxillofacial Surgery, Faculty of Dentistry, Damascus University, Damascus, SYR

**Keywords:** exodontia, dry socket, eugenol, nigella sativa oil, alveolar ostitis

## Abstract

Research background

Dry socket is one of the most common complications occurring after the extraction of a permanent tooth, but despite its high incidence, there is no established treatment for this condition. Nigella sativa oil has anti-inflammatory properties and enhances wound healing. Thus, we have decided to conduct a study to evaluate the efficacy of Nigella sativa oil in the context of dry sockets.

Aim of the study

This study aims to evaluate the effect of a Nigella Sativa oil dressing compared with an Eugenol dressing for the treatment of dry sockets in terms of accelerating soft tissue healing and reducing the intensity of inflammation.

Materials and methods

A total of 36 patients (19 males, 17 females), ranging between 20 and 50 years, 40 sockets with Alveolar osteitis randomized into 20 sockets for each group. In the first group, Eugenol with a Gelfoam carrier was used, in the second group, Nigella Sativa oil with a Gelfoam carrier was used and after copious irrigation with normal saline in both groups. Soft tissue healing and the degree of inflammation were monitored on the third (T1) and seventh (T2) days.

Results

The results of our study showed clinical and statistical superiority in favor of the Nigella Sativa oil group compared to the Eugenol group at time T2, where the P-value was less than 0.05.

Conclusions

Within the limits of our study, we found that Nigella Sativa oil led to better healing of soft tissues and reduced the intensity of inflammation in the context of dry socket, and was superior in effectiveness to Eugenol, and we recommend its use for the treatment of dry socket.

## Introduction

Alveolar osteitis (AO)/Dry socket (DS) is one of the most common postoperative complications of dental extraction [1]. Notwithstanding its high incidence, there is not a unanimous treatment for this condition [2]. AO incidence varies between 0.5% and 5% in routine dental extractions and 25% and 30% in the extraction of impacted third molars [3]. AO is usually characterized by several clinical symptoms, but pain at or near the site of tooth extraction is one of the most important symptoms [1]. The main objective of AO treatment should be pain control until normal reparative processes are initiated [4]. When evaluating this condition, the amount of exposed alveolar bone must be a factor to consider [2]. Classically, the management of AO has been irrigation, surgical interventions, and placement of medicated dressings such as Eugenol dressing. Irrigation enhances healing by removing debris, sequestra, and bacteria from the socket. Most commonly, normal saline and chlorhexidine gluconate are used [5]. The concept of AO management is changing toward alternative treatment techniques, including plasma-rich growth factors, low-level laser therapy, and other interventions such as the use of honey. Nevertheless, none of these methods have proven a 100% success ratio for prevention or treatment [6]. Medicines extracted from plants and their derivatives have great effectiveness in wound management. Besides being available and cheap, they are also considered safe as they are rarely associated with provoking allergic reactions to them [7]. Many herbal preparations have been used in wounds treatment, such as aloe vera, turmeric, sidr, and jasmine, and some have been used specifically to treat AO, such as honey and turmeric [8]. Nigella Sativa (NS) affiliates to the Ranunculaceae family and has been used in alternative medicine, both orally and topically, for a long time [9]. This herbal plant has a range of therapeutic properties, as it possesses analgesic, anti-inflammatory, antibacterial, antiviral, antifungal and antioxidant activities [10]. Medical literature mentioned that NS contains many effective compounds; foremost among them is Thymoquinone (TQ), which is included in the composition of Nigella Sativa oil, and this compound was confirmed to have many therapeutic properties [11]. In addition, Nigella Sativa and its bioactive compounds promote wounds and bone healing [12-14]. In general, the studies conducted on NS and TQ in relation to dentistry are preliminary and limited, and most of them were conducted on experimental animals. However, the results showed that the plant has promising therapeutic properties that may be useful for oral and dental diseases [15]. Based on the previous data, we decided to conduct a study to evaluate the efficacy of Nigella Sativa oil on soft tissue healing and inflammation reduction compared to Eugenol in the Context of Dry Socket.

## Materials and methods

Study design

A comparative clinical study was conducted. The study sample consisted of 36 patients suffering from Alveolar osteitis (AO) in the posterior region of the mandible who attended the Department of Oral and Maxillofacial Surgery at the Faculty of Dentistry, Damascus University. The sample included 19 males and 17 females who met the inclusion criteria and agreed to complete the study procedures and attend the follow-up sessions. The study was conducted between May 2021 and September 2022. G.power 3.1 software was used to calculate the sample size; the effect size was calculated at 1.68 (partial ŋ2 = 0.739 ) with consideration of the pilot study results using Nigella Sativa oil in 10 sockets with alveolar osteitis. F-test family was selected with ANOVA: Repeated measures within groups Test, α error prob 0.05, power 0.95, with 1 group, 3 number of measurements (T0, T1, T2), and non-sphericity correction value 1. The minimal total sample size was six samples; it was raised to 20 samples for each group. The patients were randomly divided into two groups: *Group A:* Eugenol (Kab Pharma® Syria, 100% Eugenol) was applied with a gelfoam (Cutanplast® Milano-Italy) carrier. *Group B:* Nigella sativa oil (Tact® Syria, 100% natural cold-pressed Nigella sativa oil without any synthetic additives) was applied with a gelfoam carrier. The selection of research samples was subjected to a set of criteria: Patient's age should be between 20 and 50 years, suffering from alveolar osteitis in the posterior region of the mandible after simple exodontia; the absence of general or local diseases that interfere with wounds healing, smokers and alcoholics were excluded from the study sample. The study protocol was approved by the Research Ethics Committee of Damascus University (registration no. 2021-1815).

Working methodology

A clinical examination was conducted for the patients, and AO was evaluated depending on the classic symptoms (pain, absence of the blood clot, and halitosis). The patient's compliance with the research criteria was confirmed. The patient was informed about the procedure to be applied, and his/her consent to participate in the research sample was obtained. Copious irrigation was done using normal saline (sodium chloride 0.9%), and the socket was curetted if there were any residues within it. The dressing was applied on a gelfoam carrier, and then sterile gauze was placed on which the patient would bite for half an hour. This procedure was repeated at the following times: T0: When a dry socket was diagnosed at the first visit. T1: follow-up session on the third day. T2: follow-up session on the seventh day. All the clinical procedures (clinical examination, irrigation, curettage if needed, and applying dressing) were done by the same operator (resident in the department of Oral and Maxillofacial surgery, faculty of dentistry, Damascus university). Data collection (documentation of cases and recording scores of indexes) was done by another operator (an assistant professor in the department of Oral and Maxillofacial surgery, faculty of dentistry, Damascus university) To avoid bias in the study. Both the patient and the statistician were blinded to the materials used in the study. Analgesics or any other medications were not prescribed.

Postoperative procedures 

Soft tissue healing from post-extraction AO was evaluated clinically by the coverage of the exposed bony walls with intact restorative granulation tissue; the granulation tissue formation index can be graded as [16]: 0‑ no bony walls exposed, 1‑ only one bony wall exposed, 2‑ two bony walls exposed, 3‑ three bony walls exposed, and 4‑ four bony walls exposed. The severity of inflammation was evaluated clinically by gently probing all the walls of the extraction socket with a gingival probe and checking for the presence or absence of bleeding. The degree of inflammation index can be graded as [16]: 0- no inflammation (no bleeding), 1- mild inflammation (mild bleeding), 2- moderate inflammation (moderate bleeding), 3- severe inflammation (severe bleeding). Patients were recalled for follow-up on the third and seventh day.

Statistical analysis

The data were analyzed statistically using IBM Corp. Released 2011. IBM SPSS Statistics for Windows, Version 20.0. Armonk, NY: IBM Corp., descriptive data were presented involving frequency distribution, the mean with the standard deviation, and the median. Friedman test was used to find statistically significant differences between the study times in each group, and post hoc pairwise comparisons were done by Wilcoxon signed rank test with Bonferroni corrections. Mann-Whitney test was used to find statistically significant differences between the groups at every study time; the level of significance was concluded at P < 0.05.

## Results

A total of 36 patients (19 males, 17 females) with mean 36.4 ± 9.6 years old, ranging between 20 and 50 years, 40 sockets with Alveolar osteitis randomized into 20 sockets for each group. According to the dental alveolar sites, the sample was distributed: 25% at the second premolar alveolar, 45% first molar, 10% second molar, and 20% third molar. The most frequent granular tissue index scores in group A were: 4 at T0 with 40%, 2 and 3 at T1 with 35%, and 1 at T3 with 40%. On the other hand, in group B the most frequent scores were: 4 at T0 with 65%, 3 at T1 with 35%, and 1 at T3 with 50%. The most frequent inflammation index scores in group A were: 3 at T0 with 85%, 3 at T1 with 75%, and 1 at T3 with 40%. On the other hand, in group B the most frequent scores were: 3 at T0 with 90%, 3 at T1 with 60%, and 1 at T3 with 50% (Table 1). 

**Table 1:** Frequency distribution and percentage of the healthy granulation tissue index scores and the inflammation index scores in each group.

Group	Time	Granular tissue index score				
		0	1	2	3	4
A	T0	0(0%)	0(0%)	6(30%)	6(30%)	8(40%)
	T1	0(0%)	2(10%)	7(35%)	7(35%)	4(20%)
	T2	3(15%)	8(40%)	7(35%)	2(10%)	0(0%)
B	T0	0(0%)	0(0%)	1(5%)	6(30%)	13(65%)
	T1	0(0%)	3(15%)	4(20%)	7(35%)	6(30%)
	T2	9(45%)	10(50%)	1(5%)	0(0%)	0(0%)
		Inflammatory index score				
		0	1	2	3	
A	T0	0 (0%)	1(5%)	2(10%)	17(85%)	
	T1	0(0%)	1(5%)	4(20%)	15(75%)	
	T2	3(15%)	8(40%)	7(35%)	2(10%)	
B	T0	0(0%)	1(5%)	1(5%)	18(90%)	
	T1	0(0%)	0(0%)	8(40%)	12(60%)	
	T2	8(40%)	10(50%)	2(10%)	0(0%)	

There was a decrease in the healthy granulation tissue index scores and the inflammation index scores after three days and after seven days, with statistically significant differences in each group according to the Friedman test (Table 2). 

**Table 2:** This table shows mean and standard deviation, and median with Friedman test results. Statistically significant differences between the study times in group A and group B were founded according to Friedman test P<0.05. Post hoc pairwise comparisons for T1/T2 and T0/T2 showed there were statistically significant differences according to Wilcoxon signed rank test with Bonferroni corrections in both groups when P<0.0167.

Index	Group	Time	Mean	Standard deviation	Median	Friedman Test		
						Mean rank	χ2	P value
Granular tissue	A	T0	3.10	0.852	3	2.70	37.18	P < 0.01
		T1	2.65	0.933	3	2.30		
		T2	1.40	0.883	1	1.00		
	B	T0	3.60	0.598	4	2.85	37.73	P < 0.01
		T1	2.80	1.056	3	2.15		
		T2	0.60	0.598	1	1.00		
Inflammation	A	T0	2.80	0.523	3	2.50	34.86	P < 0.01
		T1	2.70	0.571	3	2.40		
		T2	1.40	0.883	1	1.10		
	B	T0	2.85	0.489	3	2.60	35.18	P < 0.01
		T1	2.60	0.503	3	2.38		
		T2	0.70	0.657	1	1.03		

By comparing group A with group B, the granular tissue scores and inflammation scores were statistically lower in group B at T2 (Table 3).

**Table 3:** Mann-Whitney test results when comparing A with B at T0, T1, and T2. *Statistically significant differences P < 0.05.

Index	Time	Group	Median	Mean rank	Z value	P value
Granular tissue	T0	A	3	17.25	-1.938	0.053
		B	4	23.75		
	T1	A	3	19.48	-0.578	0.563
		B	3	21.53		
	T2	A	1	25.60	-2.951	0.003*
		B	1	15.40		
Inflammation	T0	A	3	19.48	-0.448	0.655
		B	3	21.53		
	T1	A	3	21.80	-0.862	0.389
		B	3	19.20		
	T2	A	1	24.95	-2.571	0.010*
		B	1	16.05		

**Figure 1:**
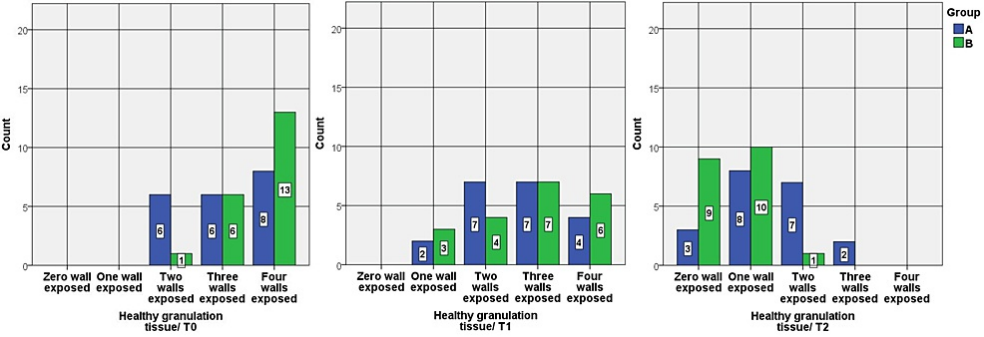
Bar charts show a comparison between the scores of the Healthy granulation tissue index between group A and group B at T0, T1, and T2. There was a statistically significant difference between A and B at T2 with a moderate size effect r = - 0.467.

**Figure 2:**
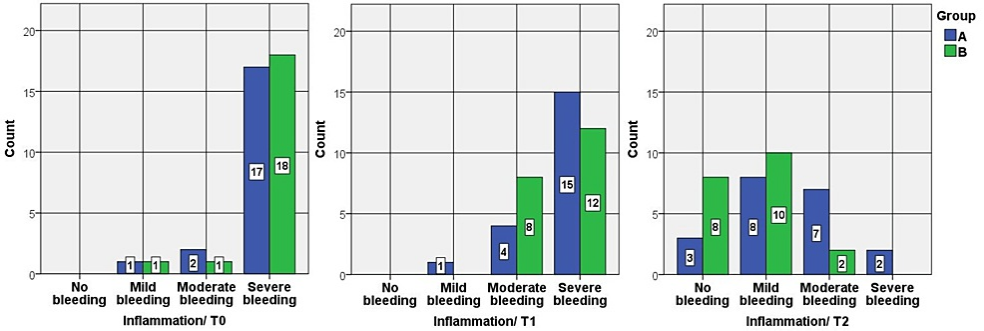
Bar charts show a comparison between the results of the inflammation index between group A and group B at T0, T1, and T2. There was a statistically significant difference between A and B at T2 with a moderate size effect r = -0.412.

In both groups, the healthy granulation tissue index scores and the inflammation index scores decreased significantly during the study times and precisely between the third day and seventh day/pre-intervention time and seventh day. However, group B was statistically significantly lower on the seventh day, and there were no statistically significant differences on the third day between the groups (Figures 1, 2).

## Discussion

The first author to define Alveolar Osteitis (AO) was Crawford in 1896; the mechanism of AO includes localized fibrinolysis within the socket, which leads to the decomposition of the blood clot, leaving the socket empty [1]. Alveolar Osteitis is usually marked by the absence of the blood clot from the socket accompanied by constant discomfort and tingling pain in and around the extraction site, usually beginning between the first and third days after extraction [17]. Apart from the fibrinolytic activity, the precise mechanism that causes AO is poorly understood [2]. The results of our study showed clinical and statistical superiority in favor of the Nigella Sativa oil group compared to the Eugenol group at time T2 (7th day), where the P-value was less than 0.05. Idrees et al. [18]; conducted a study to evaluate the efficacy of a mixture of Nigella Sativa and Salvadora persica in the treatment of AO; the study confirmed that this mixture was effective in pain relieving and inflammation reduction in the context of Alveolar Osteitis, and can be used as an alternative to Alvogel, the results of this study matches our study results. Singh et al. [12] confirmed that the direct topical application of the ethanolic derivative of Nigella Sativa by irrigation in the gingival sulcus of patients with moderate-to-severe gingivitis led to a decrease in the Gingival Index (GI) value, and therefore the use of Nigella Sativa derivatives led to better wounds healing, and it can be used as a supportive treatment for scaling and root debridement to obtain better soft tissue healing, the results of this study confirm our study results. Nordin et al. [14] conducted a systematic review to find out the effect of Nigella Sativa and its bioactive components on type 2 epithelial to mesenchymal transition (EMT); the systematic review concluded that the effect of Nigella Sativa and its active compounds, especially Thymoquinone (TQ), on EMT type2 enhanced wounds healing and reduced the rate of inflammation and fibrosis by regulating this vital process. However, further studies need to be done in order to prove these results. Dalimunte et al. [19] studied the effect of a gel derived from Nigella Sativa in post-extraction wound healing, and the results were in favor of our study results; the study concluded that the gel derived from NS contributed to accelerating the healing of the socket resulting from simple extraction. Nigella Sativa oil and Thymoquinone (TQ) showed a dose-dependent anti-inflammatory activity when tested on experimental rats, but this effect was lesser than that of aspirin [20]. The anti-inflammatory activity of TQ may be attributed to its ability to inhibit oxidizing compounds produced by arachidonic acid such as thromboxane B2 and leukotriene by blocking cyclooxygenase and lipoxygenase enzymes [21]. Khan et al. [22] confirmed that using a mixture of Nigella sativa (consisting of Nigella sativa powder and Nigella sativa oil) contributed to immediate pain relief upon application, outperforming Alvogel, which helped gradually in pain control, and the study confirmed that a Nigella sativa mixture can be used effectively in the treatment of dry socket. The study did not evaluate soft tissue healing and the intensity of inflammation. Despite the strengths of this study, such as regular follow-up of the patients, this study was met with some limitations. Firstly, the sample size of the patients in each group was small, and lastly, study materials needed a carrier (gelfoam) due to their oily nature. To the best of our knowledge, this is the first comparative study of Nigella Sativa oil to evaluate soft tissue healing and the intensity of inflammation in the context of dry socket and is aimed to find out the best and most effective medication for the treatment of this condition.

## Conclusions

Within the limits of our study, we found that using eugenol promoted wound healing and reduced the intensity of inflammation in the context of dry socket, but Nigella Sativa oil was superior in effectiveness, especially on the seventh day. This study demonstrated that the topical administration of Nigella Sativa oil was effective, and we recommend using it for the treatment of dry socket.

## References

[1] Kumar N (2020). Dry socket etiopathogenesis, management and prevention: a brief systematic review of literature. Eur J Mol Clin Med.

[2] Taberner-Vallverdú M, Nazir M, Sánchez-Garcés MÁ, Gay-Escoda C (2015). Efficacy of different methods used for dry socket management: A systematic review. Med Oral Patol Oral Cir Bucal.

[3] Kumar D, Ganapathy D, Lecturer S, Sciences T (2020). Study on occurrence and management of alveolar osteitis. PalArch’s J Archaeol Egy.

[4] Reeshma S, Dain CP (2021). Comparison of platelet-rich fibrin with zinc oxide eugenol in the relief of pain in alveolar osteitis. Health Sci Rep.

[5] Khan ZA, Prabhu N, Ahmed N (2022). A comparative study to evaluate the effect of honey and zinc oxide eugenol dressing for the treatment of dry socket: a double-blind randomized controlled trial. Appl Sci.

[6] Chakravarthi S (2017). Platelet rich fibrin in the management of established dry socket. J Korean Assoc Oral Maxillofac Surg.

[7] Lone PA, Ahmed SW, Prasad V, Ahmed B (2018). Role of turmeric in management of alveolar osteitis (dry socket): A randomised clinical study. J Oral Biol Craniofac Res.

[8] Sanchis JM, Sáez U, Peñarrocha M, Gay C (2004). Tetracycline compound placement to prevent dry socket: a postoperative study of 200 impacted mandibular third molars. J Oral Maxillofac Surg.

[9] Hussain SA, Mohammed Ameen HA, Mohammed MO, Ahmed KM, Hama-Gareb Ali R, Safar BM, Saeed KA (2019). Nigella sativa oil mouth rinse improves chemotherapy-induced oral mucositis in patients with acute myeloid leukemia. Biomed Res Int.

[10] Yimer EM, Tuem KB, Karim A, Ur-Rehman N, Anwar F (2019). Nigella sativa l. (black cumin): a promising natural remedy for wide range of illnesses. Evid Based Complement Alternat Med.

[11] Haseena S, Aithal M, Das KK, Saheb SH (2015). Phytochemical analysis of Nigella sativa and its effect on reproductive system. J Pharm Sci Res.

[12] Singh V, Gupta A, Verma UP, Mishra T, Pal M (2019). An evaluation of the efficacy of ethanolic extract of Nigella sativa L. (Kalonji) on the clinical parameters of moderate‑to‑severe gingivitis: A split‑mouth clinical study. Ayu.

[13] Ezirganli S, Kazancioglu HO, Ozdemir H, Inan DS, Tek M (2016). The effects of nigella sativa seed extract on bone healing in an experimental model. J Craniofac Surg.

[14] Nordin A, Kamal H, Yazid MD, Saim A, Idrus R (2019). Effect of Nigella sativa and its bioactive compound on type 2 epithelial to mesenchymal transition: a systematic review. BMC Complement Altern Med.

[15] Al-Attass SA, Zahran FM, Turkistany SA (2016). Nigella sativa and its active constituent thymoquinone in oral health. Saudi Med J.

[16] Sharma A, Aggarwal N, Rastogi S, Choudhury R, Tripathi S (2017). Effectiveness of platelet-rich fibrin in the management of pain and delayed wound healing associated with established alveolar osteitis (dry socket). Eur J Dent.

[17] Rakhshan V (2018). Common risk factors of dry socket (alveolitis osteitis) following dental extraction: A brief narrative review. J Stomatol Oral Maxillofac Surg.

[18] Idrees A, Abdullah N, A. Salim H, Ghanim F (2022). The validity of Salvadoria persica and Nigella sativa in the treatment of dry socket. Rev Española Cirugía Oral y Maxilofac.

[19] Dalimunte RS, Hanafiah OA, Rusdy H (2020). The effect of black cumin (Nigella sativa sp.) gel extract in wound healing process post tooth extraction. J Biomimetics, Biomater Biomed Eng.

[20] Pise HN, Padwal SL (2017). Evaluation of anti-inflammatory activity of nigella sativa: An experimental study. Natl J Physiol Pharm Pharmacol.

[21] Mansour M, Tornhamre S (2004). Inhibition of 5-lipoxygenase and leukotriene C4 synthase in human blood cells by thymoquinone. J Enzyme Inhib Med Chem.

[22] Khan ZA, Prabhu N, Ahmed N (2022). A comparative study on alvogyl and a mixture of black seed oil and powder for alveolar osteitis: a randomized double-blind controlled clinical trial. Int J Clin Pract.

